# Modeling and Simulation Approaches for Cardiovascular Function and Their Role in Safety Assessment

**DOI:** 10.1002/psp4.18

**Published:** 2015-03-11

**Authors:** TA Collins, L Bergenholm, T Abdulla, JWT Yates, N Evans, MJ Chappell, JT Mettetal

**Affiliations:** 1 Drug Safety and Metabolism, AstraZenecaAlderley Park, Macclesfield, UK; 2 School of Engineering, University of WarwickUK; 3 Oncology, AstraZenecaAlderley Park, Macclesfield, UK; 4 Drug Safety and Metabolism, AstraZenecaWaltham, Massachusetts, USA

## Abstract

Systems pharmacology modeling and pharmacokinetic-pharmacodynamic (PK/PD) analysis of drug-induced effects on cardiovascular (CV) function plays a crucial role in understanding the safety risk of new drugs. The aim of this review is to outline the current modeling and simulation (M&S) approaches to describe and translate drug-induced CV effects, with an emphasis on how this impacts drug safety assessment. Current limitations are highlighted and recommendations are made for future effort in this vital area of drug research.

Cardiovascular (CV) function is essential for life; the heart efficiently pumps blood containing vital nutrients and oxygen through vessels to cells throughout the body and removes waste products and carbon dioxide. CV function is controlled by the central nervous system, autonomic nervous system, and endocrine system, and is able to respond to a variety of external stimuli, while feedback mechanisms maintain homeostatic control. It adapts to the body's needs; for example, during exercise the heart rate (HR) increases, causing an increase in cardiac output (CO), and the proportion of CO is increased to muscles. Drug-induced CV side effects are undesirable because they may cause long-term CV damage, which puts the patient at greater risk of mortality and morbidity.

CV function declines with age: stiffened arteries lead to increased systolic arterial pressure,^1^ and a reduction in maximum HR causes a compensatory stroke volume (SV) increase in order to maintain CO during, e.g., exercise.^2^ Therefore, as the general population ages, more patients are likely to present with preexisting CV conditions, which when combined with drug-induced CV changes could result in even greater risks. Understanding and predicting the consequences of these safety changes are challenges for the development of new drugs.

Mathematical modeling of drug effects and the CV system can aid this understanding, and there are numerous examples of pharmacokinetic-pharmacodynamic (PK/PD), mechanistic, and systems pharmacology approaches in the literature. In this review, modeling analyses are explored in these three approaches defined as:

1. Traditional “top-down” PK/PD modeling and simulation (M&S) that utilizes empirical or descriptive models to describe the linkage between drug concentration and observed response, such as change in functional or structural CV biomarkers.
2. Mechanistic or systems biology “bottom-up” approaches that combine knowledge of the system from cellular targets to their impact at a cellular, tissue, or whole-body level.
3. Systems pharmacology^3^ “middle-out” approaches that sit at the interface between the other two categories. These combine aspects of both PK/PD and systems biology, and incorporates physiological processes and mechanism of action at targets.

M&S approaches promise greater impact in the CV safety space by integrating *in silico*, *in vitro*, and *in vivo* preclinical data with mechanism-based models to anticipate and predict the effects of new drugs in humans.^4^ The application of these principles is now beginning to become reality in CV safety research, although it is important not to overlook earlier empirical PK/PD models that have been used in cross-species comparison of CV PK/PD relationships. Prospective predictions of human effects at expected therapeutic doses is a vital component of preclinical CV safety risk assessment for potential new drugs and is a rapidly developing area of interest. However, this is only valuable if clinical data can be obtained and compared to the model-based predictions. If differences between predicted and observed magnitude and kinetics of drug-induced effects can be better understood, then progress in refinement of prospective predictions of CV safety endpoints can be achieved.

## OVERVIEW OF DRUG-INDUCED CARDIOVASCULAR EFFECTS

**Figure 1:**
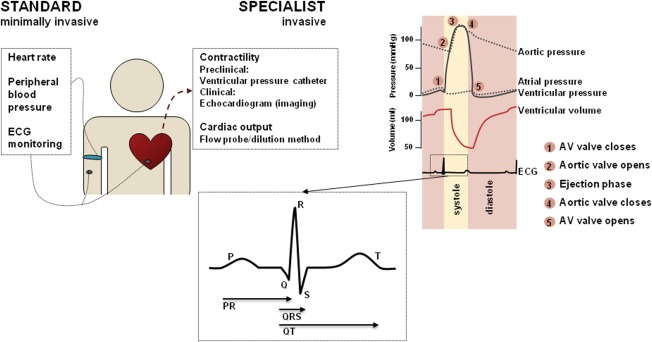
Standard minimally invasive and invasive CV measurements that can be obtained from a whole body system. The Wiggers diagram (top right) shows the dynamics of some measurable variables, including atrial and ventricular pressure as well as ventricular volume. Also, a standard 12-lead ECG curve is shown, and the main intervals (QT, QRS, and PR) indicated.

A wide panel of electrophysiological and mechanical functional markers is collected during clinical and preclinical *in vivo* testing. These are outlined in **Figure** 1 and typically include:

1. Electrocardiograph (ECG) measurements. Common ECG interval measurements include QT interval, QRS duration, and PR intervals, all measured in milliseconds (ms). The QT interval is often corrected for HR, defined as corrected QT (QTc).
2. Hemodynamic measurements including HR and blood pressure (BP), measured in beats min^-1^ and mmHg, respectively.
3. Indices of contractility including the maximum and minimum rate of left ventricular pressure change (dP/dt max (+) and dP/dt min (–)), measured in mmHg s^-1^, and QA interval, which is an inverse index measured in ms (time between Q wave on ECG and onset of arterial wave (blood pressure)). Ejection fraction (EF) is a common clinical biomarker measured as the percentage of the diastolic volume of blood in the ventricle that is ejected with each beat.
4. In preclinical pathological studies, chronic changes such as histopathology of the heart and/or vessels are also monitored.

Functional changes of ECG and hemodynamics, although not typically life-threatening in themselves, are readily monitorable, although they may be associated with more serious but rare events. Most famously, QT/QTc prolongation is known to be a risk factor for the potentially fatal arrhythmia Torsades de Pointes (TdP), which caused one-third of drug withdrawals between 1990 and 2006.^5^ In addition, prolongation of the PR and QRS intervals are linked to CV mortality and morbidity in cardiac risk populations.^6^ HR and BP changes are known to increase risk to all causes of mortality, including CV complications.^7^,^8^ Contractility changes typically require measurements through more technical or invasive techniques and are therefore more difficult to obtain clinically, but can lead to heart failure, hypertrophic cardiomyopathy, and sudden cardiac death.^9^ Other examples of cardiac safety liability include the withdrawal of rofecoxib, a selective Cox-2 inhibitor, from the market after long-term use showed increased risk of serious thrombotic events including myocardial infarction and stroke.^10^

Cardiovascular complications were the leading cause of drug withdrawals from the EU market during 2002 and 2011^11^ across a range of therapy areas. Any unintended drug-induced effects on the CV system represent a potential safety concern, and in order to avoid costly late-stage failures, a variety of early preclinical experiments were performed *in vitro*, *in silico,* and *in vivo* to rule out unsafe compounds or to understand and quantify risk with those that do progress. Novel therapeutics must distinguish themselves from standard of care agents either in terms of improved efficacy or safety risk, so a thorough assessment of CV effects is essential.

Preclinical Safety studies with CV endpoints include:

1. “Off-target” activity assessed *in vitro* on molecular targets (enzymes, GPCRs, ion channels) associated with effects on CV structure and function.
2. *In silico* and quantitative structure–activity relationships (QSAR) models built using data from such *in vitro* “off-target” data.
3. *In vitro* functional assessments using traditional tissue-based studies (e.g., isolated heart/blood vessel pharmacology), supplemented with new technologies such as human stem cell-based cardiomyocytes.
4. *In vivo* functional assessment is mainly run in conscious animals and measures ECG, BP, and HR via implantable telemetry in single-dose, safety pharmacology studies at the therapeutic range and above.^12^
5. *In vivo* assessment of the effects on CV structure in repeat-dose toxicology studies assessing clinical pathology, histopathology, and clinical observations. Functional assessments of HR and ECG can also be made in such studies using jacketed telemetry.

All of these endpoints contribute to an integrated CV risk assessment of the drug before it is first administered to humans. In the following sections we cover modeling approaches applied to drug-induced effects on ECG intervals, hemodynamics (including contractility), and cardiac damage, and where appropriate, highlight examples of translation from preclinical to humans.

## DRUG-INDUCED CHANGES ON ECG INTERVALS

The ECG reflects the electrical depolarization and repolarization that cardiomyocytes undergo during an action potential (AP). It represents a combined view of the spread of excitation that occurs across the cardiac tissue. Changes in ECG intervals can indicate changes in cardiac electrophysiology, for example, resulting from ion channel inhibition.

One of the most frequently assessed ECG intervals is QT, and with increasing concern regarding QT/QTc prolongation, the ICH announced in 2005 preclinical (S7B)^13^ and clinical (E14)^14^ regulatory guidance for new drugs. These focused on QT prolongation and blockade of the ion channel (Kv11.1) encoded by the human ether-a-go-go-related gene (hERG). The E14 guidelines states that in thorough QT/QTc (TQT) studies, the threshold level of regulatory concern is at 5 ms, as evidenced by an upper bound of the 95% confidence interval (CI) around the mean effect on QTc exceeding 10 ms.^15^ PK/PD modeling of QTc prolongation has been beneficial in this setting for predicting QTc prolongation at doses that were not directly studied in the TQT study and to support statistical analysis.

Biomarkers such as QT prolongation are sensitive, but not specific, predictors of ventricular proarrhythmia (i.e., TdP), which can be complicated by further mechanisms, for example, multiple ion channel blockade or effects on trafficking of the ion channel to the cell membrane. PK/PD modeling of QT prolongation began in the late 1970s (see **Table** 1 and **Supplementary Material** for references). Arguably, this is the most characterized of all PK/PD relationships of ECG interval changes due to the regulatory focus on the link between hERG, QT, and TdP. In earlier reviews of drug-induced QT effects,^16^,^17^ the following factors have been identified as important to consider during the modeling of QT, but could also remain relevant for other ECG endpoints:

1. Heart rate correction, preferably individual-specific.
2. Variability of baseline, both interindividual and intraindividual, as in circadian rhythm.
3. Subject demographic information such as age, sex.
4. Genetics, e.g., rare polymorphisms causing long QT syndrome.
5. Environmental or other factors such as obesity, physical activity, electrolyte levels, blood pressure, blood glucose, and alcoholism.

**Table 1:** Overview of the composition of PK/PD models used for modeling of ECG intervals in preclinical species and human, indicating selected concentration–effect relationship, model for capturing potential time delays and baseline function

Variable	Species	Concentration–effect relationship	Baseline function	Time delay model
QTc	Human	Linear: disopyramide,^1^,^2^ quinidine,^3^–^5^ sotalol,^6^–^8^ N-acetylprocainamide,^9^ sematilide,^10^ dofetilide,^11^ citalopram,^12^ lamotrigine,^13^^a^ prulifloxacin,^14^^a^ moxifloxacin,^8^,^13^,^14^ grepafloxacin,^8^ mifepristone,^15^^a^ cabazitaxel,^16^^a^ AZD1386,^17^ NCE01-03^18^ Exponential: AZD3839^19^ E_max_: disopyramide,^20^ N-acetylprocainamide,^9^ sematilide,^10^ azimilide,^21^ AZD1305^17^ E_max_ sigmoidal: dofetilide,^11^ vernacalant^22^ Operational agonism: dofetilide^23^	Constant: sotalol,^3^,^7^ sematilide,^10^ dofetilide,^11^ citalopram,^12^ mifepristone,^15^ cabazitaxel,^16^ azimilide,^21^ AZD1305,^17^ vernacalant^22^ 1x cosine: sotalol,^8^ moxifloxacin,^8^ grepafloxacin,^8^ NCE01-03^18^ 3x cosine: AZD3839^19^ Unknown cosine: lamotrigine,^13^ moxifloxacin,^13^ AZD1386^17^	Effect compartment: disopyramide,^1^ quinidine,^3^,^4^ sotalol,^7^ N-acetylprocainamide,^9^ sematilide,^10^ dofetilide,^11^ citalopram,^12^ AZD1305,^17^ AZD3839^19^
QTc	Rat	Linear: quinidine,^24^ roxithromycin,^25^ azithromycin^25^ E_max_: terfenadine,^24^ clarithromycin,^25^ erythromycin,^26^ ebastine^27^		Effect compartment: roxithromycin,^25^ azithromycin,^25^ clarithromycin,^25^ erythromycin,^26^ ebastine^27^
QTc	Guinea pig	Linear: Imipramine,^28^ fluvoxamine^28^ E_max_: tacrolimus^29^		Effect compartment: Imipramine,^28^ fluvoxamine^28^ Myocardial compartment: tacrolimus^29^
QTc	Dog	Linear: AZD1305,^17^ AZD1386,^17^ cisapride,^30^ sotalol,^30^ moxifloxacin,^30^ Compound 2,^31^ quinidine,^32^ NCE01-03^18^ E_max_: AZD3839^19^ E_max_ sigmoidal: dofetilide,^33^ cisapride,^34^,^35^ moxifloxacin,^34^ terfenadine,^35^ E-4031^35^	Constant: cisapride,^34^,^35^ moxifloxacin^34^ Linear: dofetilide^33^ Unknown cosine: AZD1305,^17^ AZD1386^17^ 1x cosine: cisapride,^30^ sotalol,^30^ moxifloxacin,^30^ NCE01-03^18^ 3x cosine: AZD3839^19^	Effect compartment: AZD1305,^17^ quinidine,^32^ dofetilide,^33^ cisapride,^35^ terfenadine,^35^ E-4031,^35^ AZD3839^19^
QTc	Monkey	Linear: Compounds 1,8,9,^31^ Moxifloxacin^36^		Model C_max_-E_max_: Compounds 8–9^31^
QRS	Human	Linear: quinidine,^3^,^5^ cabazitaxel,^16^^a^ flecainide^37^,^38^	Constant: cabazitaxel,^16^ flecainide^37^,^38^	Effect compartment: quinidine^3^
QRS	Dog	Linear: R1551^39^ E_max_ sigmoidal: Compounds 3–7^31^ HR-dependent: flecainide^40^	Constant: R1551^39^ HR-dependent: flecainide^40^	Effect compartment: R1551^39^
QRS	Monkey	Linear: R1551	Constant: R1551^39^	
PQ	Human	Linear: quinidine^3^		Effect compartment: quinidine^3^
PR	Human	Linear: cabazitaxel^16^^a^	Constant: cabazitaxel^16^	
PR	Dog	Linear: R1551	Constant: R1551^39^	Effect compartment: R1551^39^
PR	Monkey	Linear: R1551	Constant: R1551^39^	Effect compartment: R1551^39^
ERP	Rabbit	Exponential: AZ13395438,^41^ Compound A and 2f^42^	Constant: AZ13395438,^41^ Compound A and 2f^42^	Effect compartment: AZ13395438,^41^ Compound A and 2f^42^

a Studies where no ECG effect was found. References contained in Supplementary Material.

ERP, effective refractory period.

QT interval duration is strongly dependent on HR and the use of correction methods aim to remove the influence of heart rate, providing a more stable measure: QTc. HR correction formulas include linear and fixed exponential (Bazett, QTcB, Fridericia, QTcF, and individual, QTcI). QTcI and QTcF have been shown to perform best in humans, while QTcI performed best in preclinical species including dog, guinea pig, and cynomolgus monkey.^18^ Cosine functions^19^ have been used to account for within-subject variability in baseline due to homeostatic mechanisms, external factors, or circadian rhythm (regular diurnal fluctuation), either using typically single or where necessary multiple cosine functions.^20^,^21^ In one example of QTc prolongation modeling, the model structure utilized three baseline cosine functions with time periods of 4, 8, and 24 hours in the dog, and 2, 4, and 24 hours in human, combined with effect compartment models for both species and for the concentration–response relationship an E_max_ model for the dog and a linear model for human.^22^

PK/PD modeling efforts have focused on QT and hERG inhibition, but there are other examples of other mechanisms affecting ECG intervals, **Table** 1 describes the type of structural models used in the descriptive PK/PD modeling of drug-induced ECG interval changes for both preclinical and clinical studies. Currently, PK/PD modeling of clinical or nonclinical data is routinely modeled using population (mixed effects) approaches rather than individual or pooled datasets.^22^,^23^ Interindividual variability is often included on baseline parameters and drug effect. This reflects the availability of high-quality, rich ECG data combined with more readily available software to conduct these analyses.

Linear, E_max_, and sigmoidal E_max_ models have all been used to describe the relationship between concentration and effect for ECG intervals.^19^ 58% of the compounds listed could be adequately described with simple linear drug effect models. While it is expected that drug effects will eventually saturate with exposure, linear models may be more prevalent for ECG changes, as maximal effects are often not achieved in safety studies where doses are selected based on margins to therapeutic exposure not to characterize the full concentration–response relationship. Studies can also be halted before reaching a maximum level due to lack of tolerability at these exposures. However, E_max_ or sigmoidal E_max_ models are often used with antiarrhythmic compounds when these exposures are tolerated. Once in the clinic, concentrations required to reach maximal effect are less often reached for these same reasons, leading to even fewer saturable models being observed.^24^ Where E_max_ models have been utilized for clinical QTc changes, the maximal activity level appears to be compound-specific rather than reaching a system-specific or physiological upper limit (for example, 20 ms for vernakalant^25^ and 170 ms for N-acetylprocainamide^26^).

It is very common to observe a delay between blood or plasma drug concentration and CV effect, resulting in hysteresis observable on a concentration–effect plot. This time delay is often short, on the order of minutes. Under these circumstances a link, or effect compartment, model is often applied,^19^ describing the delay between the measured plasma concentrations and the distribution of drug to the effect site using a single rate constant, k_e0_. Thus, 43% of compounds included a time delay, 89% of these used an effect compartment model. For ECG intervals the observed time delay rarely exceeds a half-life of 30 minutes, although two compounds, AZD3839 and AZD1305, had time delays that exceeded 100 minutes in the dog.^20^,^22^ Notably these compounds were also modeled in human and delayed effect was not as apparent, only 10–15 minutes. The use of indirect response models^19^ that describe the physiological turnover of the response parameter in terms of synthesis and degradation is uncommon for ECG intervals. One potential reason for this is that the effect of the drug on ion channel activity is expected to be rapid once the compound has reached the myocytes, not requiring a turnover process to have an effect. In addition to delays in reaching the site of action, another potential cause of observed time delays could be the production of an active metabolite.^27^

Although PK/PD modeling examples of intervals other than QT are limited, it appears most other endpoints are typically treated similar to QT, although heart rate correction may not always be required for other ECG intervals. While not as well studied, CV complications are still potentially hazardous with these endpoints. Cav1.2 inhibition leads to increased PR interval and is linked to AV block, while Nav1.5 inhibition leads to increased QRS duration and is linked to ventricular tachycardia. The concentration–QRS relationship of a number of compounds has been investigated in dog^23^ using a population approach, and this enabled comparison across compounds and investigation of the therapeutic window. The data were modeled as percent change from baseline and the size of the estimated E_max_ for QRS change varied from 8 to 57%.

In contrast to the descriptive PK/PD modeling approaches, drug-induced ECG effects have also been modeled using detailed, “bottom-up” mechanistic models. Drug effects on ion channels are described mathematically to predict morphology changes in the AP or ECG. Cellular cardiac AP models have been developed for different species including human,^28^–^31^ dog,^32^ guinea pig,^33^ and rabbit.^34^ These models represent the relevant electrophysiological aspects of the cellular system: transmembrane conductance, ion channels and their inhibition by drugs, as well as other pumps/exchangers and intracellular ion concentrations, and integrate the influence of these factors over time on cellular ion concentrations. Drug effects are modeled by altering the ion conductance term, which represents the gating (open/closed, etc.) of the relevant ion channel.

Such cardiac AP models have been applied to predict the effects of antiarrhythmic drugs that alter ion channel activities^35^–^37^ and better describe the effects of multiple ion channel inhibition than focusing purely on potency values. Davies *et al*.^35^ calibrated the Hund-Rudy canine AP model^32^ to predict change in AP duration in dog cardiomyocytes solely from *in vitro* data of five ion channels (Nav1.5, Cav1.2, Kv4.3, Kv7.1, and Kv11.1), demonstrating a predictivity of 68% (ratio of sum of true positive and true negative results to total number examined). Similarly, Mirams *et al*.^37^ showed that a human AP model could be trained to classify TdP risk based on predicted therapeutic C_max_ and Nav1.5, Cav1.2, and Kv4.3 channel median inhibitory concentration (IC50) values with markedly improved accuracy compared to safety margins between hERG IC50 and therapeutic C_max_ alone. A recent comparison of predicted effect of ion channel block in AP models from human and preclinical species highlights the importance of cautious extrapolation between species.^38^ For example, a 70% block of the hERG ion channel resulted in an 80% AP prolongation in humans but only a 30% and 20% change in dog and guinea pig, respectively.

**Figure 2:**
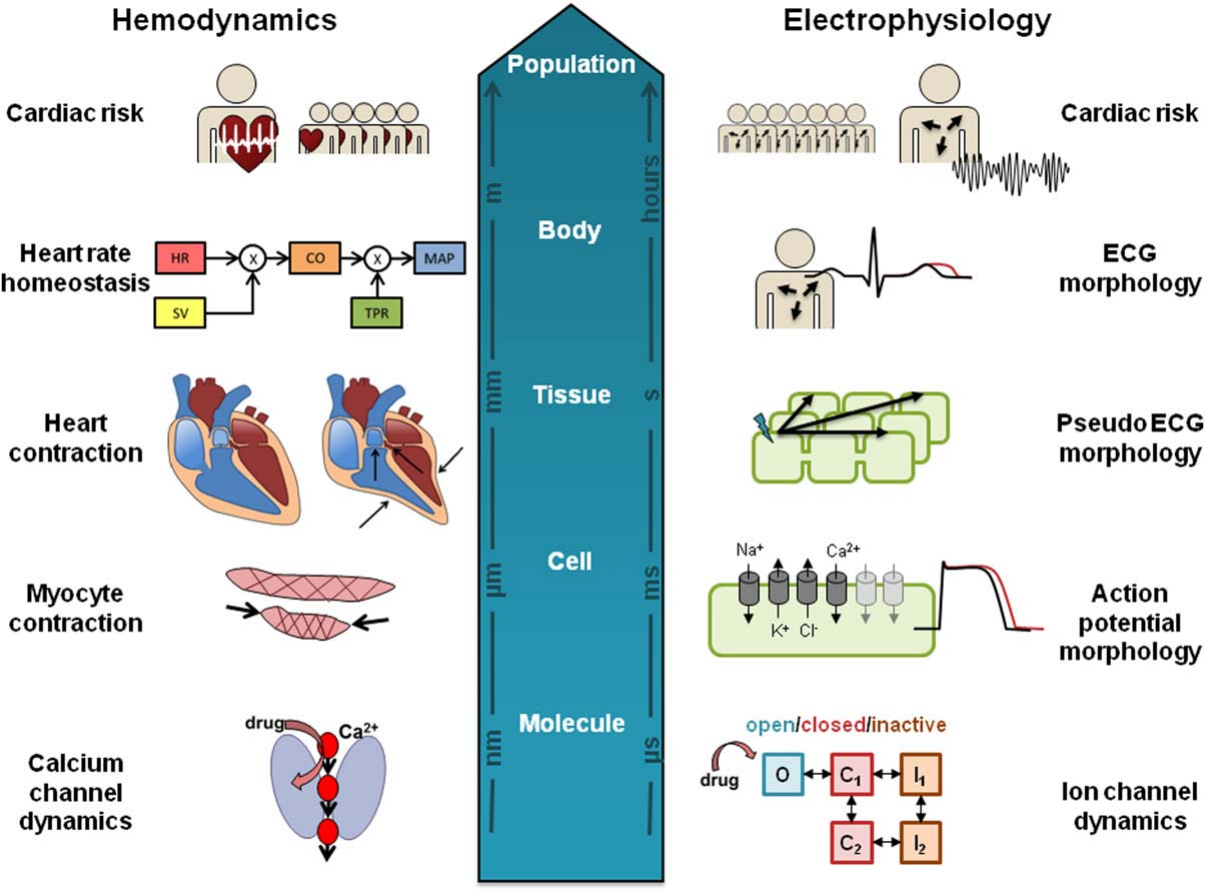
Spatial and temporal scales at which cardiovascular systems models operate. At each level, different assumptions and variabilities need to be addressed, to meet the ultimate goal of predicting cardiovascular liabilities in patient populations.

While these ion flux models can capture information relating to the membrane potential in a single cell, the resulting ECG are at the tissue/whole-body level (**Figure** 2) and depend in part on the spatial orientation of myocytes in the heart and the AP propagation in tissue. Models of cardiac tissue have therefore been constructed to describe the propagation of the AP in 1, 2, and 3 dimensions^39^ by linking multiple cellular models in a spatially relevant way. These tissue models have been used to study the effects of single and multiple ion channel blockade on Purkinje fibers.^40^ Sotalol-induced effects^41^ have been studied via pseudo-ECG generated from the simulation of a one-dimensional fiber representing proportional distribution of cells of the epicardium, midmyocardium, and endocardium. At the whole-heart level, cardiac structure and electrophysiology have been integrated with whole-body geometry to translate ion channel effects through simulation of cardiac AP propagation to calculate 12-lead ECG and QT prolongation as measured in the torso.^42^ A 12-lead ECG was also calculated by Wilhelms *et al*.^43^ for comparison of the two QTc-prolonging drugs cisapride (proarrhythmic) and amiodarone (antiarrhythmic), identifying the effect of amiodarone alone on AP conduction as the mechanism behind the drug being anti- rather than proarrhythmic.

The usefulness of mechanistic *in silico* models in drug discovery and development now needs to be demonstrated, as a recent study^44^ that investigated the ability of AP models to predict the QT change in TQT studies and showed the models in general underpredicted the TQT outcome. These became more reliable predictions of TQT outcome when the comparison range was relaxed to within a 100-fold range of concentration reached in the TQT study, suggesting simple concentration comparisons between modeled AP changes and *in vivo* plasma exposure–response requires improvement by considering other factors such as tissue distribution and intracellular concentrations.

One tool built specifically to do this is the Cardiac Safety Simulator (CSS) (Certara), a commercially available tool designed to increase the ability of nonmodelers to test the effect of ion channel activity on the ECG incorporating population variability on both exposure (through SimCYP, Certara) and ECG prediction. As a test of predictivity of the simulator, the QTc effects of six antipsychotic drugs^45^ were investigated using the CSS, showing good agreement between predicted and observed mean QTc change, although the predictions did not account for all of the observed variability. This approach represents a bottom-up approach based purely on *in vitro* data and it is potentially tempting to adopt this as a predictive strategy but not enough is yet known about the predictive performance of this tool. With a vast number of inputs and settings in such a model, this should be used cautiously until more validated examples are demonstrated, and it is hoped this will explore more diverse situations such as mixed ion channel inhibition, combined effect of parent and metabolite, and mechanisms not caused by ion channel inhibition.

In our experience AP changes can be routinely predicted once *in vitro* ion channel inhibition assay data are generated, forming a key part of the early risk assessment prior to *in vivo* data being generated. During the drug discovery process ion channel inhibition liability can often be reduced or removed via structural modification of potential drug entities, meaning only small changes will be observed *in vivo* or only at high safety margins. PK/PD modeling of *in vivo* ECG changes is then applied wherever statistically significant changes are observed. This PK/PD modeling then defines the underlying concentration–response relationship and better quantifies safety margins. If a compound is then selected to enter clinical studies, a prediction will be made at expected therapeutic doses in human combining the predicted human PK and the PD model including any time delays to assess the expected magnitude of effect.

While mechanistic modeling provides a powerful lens, empirical PK/PD modeling will still remain a valuable approach to quantifying the concentration–effect relationship of ECG intervals from both the clinical and preclinical studies in the near future, because it is an effective way to assess CV safety risk and the models are straightforward to implement with little mechanistic understanding. Mechanistically based models of ECG change have focused primarily on ion channel inhibition as the typical mechanism of effect, but due to early *in vitro* safety screens it is likely that in the future observed *in vivo* effects on ECG intervals will be small, and may be driven by other, more unusual mechanisms. While much effort has been invested in the development of mechanistic models that replicate the time course of ECG changes, it is unclear how and at what point these will be used in the drug development process. *In vitro* data becomes available early in drug evaluation, when there is limited understanding of likely exposure in human. Later, once the human dose and pharmacokinetic predictions are better defined and one could use the mechanistic model of ECG change, the *in vivo* CV results become available and could take precedence over the *in vitro-*based prediction in the integrated risk assessment.

## DRUG-INDUCED CHANGES ON HEMODYNAMICS

Hemodynamics is the study of blood circulation, which is governed by pressures and resistances in different parts of the cardiovascular system, as well as the force and rate of contraction of the heart. Typically, HR and BP are monitored including mean arterial, diastolic, and systolic blood pressure (MAP, DBP, and SBP, respectively). Other important variables include stroke volume (SV), cardiac output (CO), total peripheral resistance (TPR), contractility, and in some cases compliance and central venous pressure. The anatomy and physiology of the CV system result in fundamental interrelationships existing between these variables, including that CO is the product of HR and SV, MAP is the product of CO and TPR and others; for example, HR and contractility are known to be highly correlated *in vivo*.^46^ Despite this understanding, PK/PD modeling of drug effects is often conducted separately on individual parameters.^23^

**Table** 2 describes the application of PK/PD models to blood pressure, heart rate, and contractility effects. Baseline functions are widely used to explain the inherent variability and circadian rhythm, such as cosine functions. 76% of compounds adopt indirect response models to describe the time delay between concentration and effect, rather than a simple effect compartment model more commonly used for ECG intervals. In contrast to the observed time delays with ECG intervals, the time delays on hemodynamic parameters are far more varied, and half-lives of the indirect response parameter k_out_ could be within a few minutes to greater than 50 days. This is likely to reflect the variety and timescales of mechanisms by which a compound can affect hemodynamics. The use of indirect response models may better capture biological processes downstream of receptor activity, but upstream of CV function, for example, nitric oxide production. The underlying exposure response models most typically include linear or E_max_/sigmoidal E_max_ pharmacodynamic models.

**Table 2:** Overview of the composition of PK/PD models used for modeling of hemodynamic parameters in preclinical species and human, indicating selected concentration–effect relationship, model for capturing of potential time delays and baseline function

Variable	Species	Concentration–effect relationship	Baseline function	Time delay
BP (including models of MAP, SBP, and DBP)	Human	Linear: eprosartan,^43^ prazasin,^44^ trimazosin,^44^ doxazosin,^44^,^45^ E7080^46^ E_max_: moxonidine,^47^ fimasartan,^48^ E_max_ sigmoidal: Remifentanil^49^	Constant: Remifentanil,^49^ prazasin,^44^ trimazosin,^44^ doxazosin,^44^,^45^ E7080,^46^ nifedipine^50^ 2x cosine function: eprosartan,^43^ fimasartan,^48^ moxonidine^47^	Effect compartment: Remifentanil,^49^ eprosartan,^43^ prazasin,^44^ trimazosin,^44^ doxazosin^44^,^45^ IDR: E7080,^46^: fimasartan^48^
BP (including models of MAP, SBP, and DBP)	Rat	Linear: milrinone^51^ Log-linear: metabolites^52^ E_max_/I_max_: L-NAME,^51^ doxazosin^51^ Sigmoidal E_max_: IIDN,^52^ IMDN,^52^ ISDN^52^	Constant: IIDN,^52^ IMDN,^52^ ISDN^52^ Sällström baseline^53^: L-NAME,^51^ milrinone,^51^ doxazosin^51^	IDR: L-NAME,^51^ milrinone^51^
BP (including models of MAP, SBP, and DBP)	Guinea pig	E_max_/I_max_: milrinone,^51^ Doxazosin,^51^ L-NAME^51^	Linear: L-NAME,^51^ milrinone,^51^ doxazosin^51^	IDR: milrinone^51^
BP (including models of MAP, SBP, and DBP)	Dog	Linear: milrinone,^51^ Compound 12,^31^ doxazosin^51^ E_max_/I_max_: L-NAME^51^	Constant: Compound 12^31^ Cosine function: L-NAME,^51^ milrinone,^51^ doxazosin^51^	IDR: L-NAME,^51^ milrinone,^51^ Compound 12^31^
HR	Human	Linear: PF-00821385^54^ E_max_/I_max_: PF-00610355,^55^ cilobradine^56^	Negative Gaussian function: PF-00610355^55^ Indirect periodic function with exercise: cilobradine^56^ Cosine function: PF-00821385^54^	Effect compartment: PF-00610355^55^ Transduction: cilobradine^56^
HR	Rat	Linear: milrinone^51^ E_max_/I_max_: L-NAME,^51^ doxazosin^51^ Operational model of agonism: CPA^57^	Constant: CPA^57^ Sällström baseline^53^: L-NAME,^51^ milrinone,^51^ doxazosin^51^	IDR: L-NAME,^51^ doxazosin,^51^ milrinone^51^
HR	Guinea pig	E_max_/I_max_: milrinone,^51^ doxazosin^51^ L-NAME^51^	Linear: L-NAME,^51^ milrinone,^51^ doxazosin^51^	IDR: milrinone,^51^ doxazosin^51^
HR	Dog	Linear: milrinone,^51^ PF-00821385^54^ doxazosin^51^ Threshold linear: Compound 10,^31^ E_max_/I_max_: L-NAME^51^	Cosine function: L-NAME,^51^ milrinone,^51^ doxazosin,^51^ PF-00821385^54^	IDR: L-NAME,^51^ milrinone^51^
dP/dt max	Dog	Linear: Compound 11^31^	Constant: Compound 11^31^	IDR: Compound 11^31^

References contained in Supplementary Material. IDR, indirect response model.

HR changes were observed with an anti-HIV agent with κ-opioid agonist activity.^47^ In the dog and human studies, effects were modeled using a single cosine function for baseline daily variation and a direct linear drug effect. The mean drug effect slope was 2.3-fold steeper in dog compared to that observed in human and therefore the prediction of human response was made by using the dog slope and combining it with human PK, which slightly overestimated the clinical HR change. As a predictive strategy this is a sensible approach and represents a worst-case scenario, although in order to assess applicability across a range of mechanisms additional drugs would need to be assessed in a similar manner in order to ascertain if there are any consistencies in cross-species differences in drug effect for HR.

Since many hemodynamic measurements result from interacting mechanical and physiological processes, examples of mathematical descriptions of hemodynamics have been constructed with a systems approach in mind. These include the regulation of CO by the peripheral tissues of the body^48^ and the 2-element Windkessel model,^49^ which describes the heart and circulation as a closed system circuit, including a pump and chamber containing a pocket of air.

**Figure 3:**
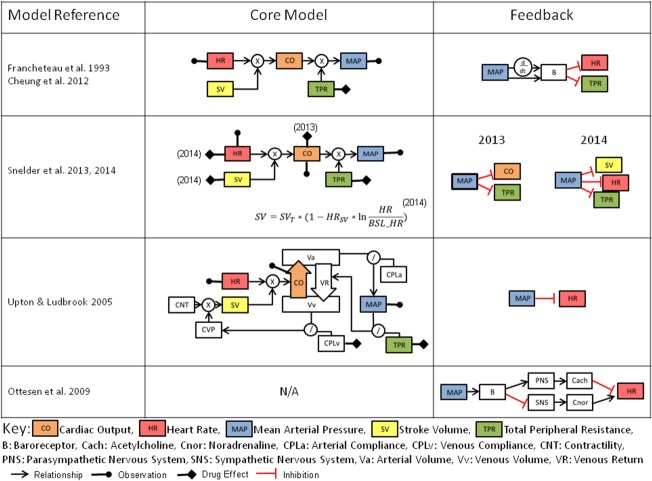
Comparison of existing hemodynamic systems pharmacology model structures including inter-relationships between variables and feedback structure. References contained in Supplementary Material.^66^–^70^

A computational physiological model was published in 1972 by Guyton *et al*.^50^ describing long-term blood pressure and cardiac output control and was later expanded.^51^ These modeling works were instrumental in illustrating the role of the renal system in long-term fluid balance and BP control. More recent efforts to develop systems pharmacology models (**Figure** 3) have generally focused on shorter-term drug effects, and therefore attempt to combine the relationships between HR, BP (also incorporated as mean arterial pressure, or MAP), CO, TPR, and SV, and can be further expanded to include contractility and compliance. Homeostatic feedback from MAP is most commonly implemented on HR, CO, SV, and via sympathetic activity to vasculature and is analogous to the mechanism of baroreceptor feedback, which regulates arterial pressure.

One such example has been applied to the clinical effects of nicardipine and nifedipine,^52^ which are L-type calcium channel blockers. The model introduces an additional feature by capturing this feedback through dual mechanisms of the proportional and rate-sensitive functions of MAP. Since only HR and MAP are typically observed, while the model includes additional state variables for CO, TPR, and SV, the system suffers structural identifiability issues. A more recent structural identifiability analysis^53^ resulted in a parameter reduction of the model, resolving this issue.

Systems pharmacology models have been applied in rat^54^,^55^ that try to resolve the identifiability issues by the monitoring of CO during the model building process. In this approach data from six compounds were combined in order to estimate the rat model parameters. The advantage of these types of models is the ability to determine the site of drug effect, and these have so far been applied to TPR, HR, SV, and CO, although notably direct drug effects on contractility and BP have not been studied in a mechanistic manner.

These hemodynamic models only account for total CO and MAP; however, the blood pressure profile (**Figure** 1) contains much more information about the heart contraction as driven by the action potential. Calcium plays a critical role in modulating contraction^56^: its cellular influx following depolarization is an indirect activator of myofilaments (**Figure** 2). This process is sensitive to myofilament stretching as the heart fills with blood, resulting in a stronger contraction, and is an important autoregulatory mechanism. Cellular models have therefore been used in describing this interplay between the kinetics of calcium gradient and the dynamics of myocyte contraction^57^–^59^ as well as their control via the autonomic nervous system. Adrenergic and muscarinic receptors mediate this process, and drugs can be antagonists at these receptors which may cause changes in indexes of contractility or other effects, such as beta-blockers (beta1 adrenoreceptor antagonists), which are used in the treatment of hypertension. To further link AP with hemodynamics, models have been produced describing the electromechanics of the whole heart,^60^,^61^ and these have combined cell excitation/contraction (EC) coupled with heart mechanics, system circulation,^62^ and autonomic control.^63^,^64^ While great progress has been made defining multiscale systems approaches (**Figure** 2), these have not yet been fully utilized for linking drug exposure with cardiovascular changes for the assessment of safety or efficacy.

In our experience, modeling hemodynamic changes for safety assessment often consists of two stages depending on the questions to be addressed. First application of a top-down PK/PD approach to the observable of greatest concern allows us to quantify the effects and reveals the steady-state concentration–response relationship. With this information in hand, one can either assess the margin between projected efficacious and safe exposures or use predicted human pharmacokinetics to generate a predicted magnitude of response at therapeutic doses. PK/PD modeling of hemodynamic parameters has been demonstrated successfully and is useful for studies when only a single hemodynamic parameter changes over time and when little or none is known about the underlying mechanism.

Second, existing systems pharmacology models can be applied to HR and BP data to provide insight into the mechanism of drug effect and the effects on the system as a whole. If system parameters already exist,^54^ we can fix these and only vary the drug-specific properties. When modeling species without preexisting systems parameters, we may need to develop a unique set of system parameters prior to using the system or modify the system parameters from other species. There is not yet feedback from the clinic to understand how successful these systems approaches are in clinical predictions.

In the future it is expected that systems pharmacology models will incorporate the simple interrelationships between BP and HR, and may overcome the identifiability issues experienced in some existing models. There will also be future opportunities to explore bottom-up approaches that have not been applied to drug-induced changes in hemodynamics.

## LINK BETWEEN ACUTE FUNCTIONAL EFFECTS AND CARDIAC DAMAGE

Long-term damage or risk of CV failure is often associated with perturbations of the cardiovascular parameters reviewed above. Structural damage can arise as a consequences of direct drug toxicity (such as necrosis of heart tissue including the valves of the heart), but can also be an indirect consequence of drug-induced dysfunction of hemodynamic or ion channel effects over time. These effects, however, are often poorly characterized. For example, even the link between concentration, QTc prolongation, and TdP, the incidence of drug-induced TdP in all patients taking the drug is likely to be very low and is not well quantified, with less than 4% of all TdP reporting fatalities.^5^ Other forms of cardiovascular damage may also have similarly low incidences of life-threatening or fatal events, and are equally difficult to link back to exposure of the drug.

Cardiotoxic agents, which are characterized by dysfunction of cardiac or vascular smooth muscle, can cause^65^:

1. Myocardial infarction
2. Venous thromboembolism
3. Cardiac arrest
4. Necrosis (e.g., cocaine)
5. Valve damage^66^

Most analyses of the risk of cardiovascular damage and other events concentrate on environment or diet-induced effects and markers of disease progression (**Table** 3). However, there are a number of examples of statistical analyses associating drug use with cardiovascular changes. Almost all of these studies considered treatment by a particular drug, or the actual dose level, and in no case were PK- or concentration-dependent effects explored. These studies also tended to be long-term and/or retrospective across multiple drugs, and so PK analysis was most likely unfeasible. These were frequently retrospective studies and so exact dose levels were also probably missing, explaining the lack of detailed exposure-driven analysis. Systems approaches to cardiovascular biomarkers for heart failure are now being recognized,^67^ although there are no examples known to date that link such biomarkers to PK/PD modeling. This is potentially an important application of systems modeling to enable the discovery of cardiovascular damage signature based on blood-borne biomarkers: Many markers of cardiovascular disease suffer, on their own, from a lack of specificity versus sensitivity.

**Table 3:** Mathematical approaches to predict cardiac damage

Drug class	Analysis method	Endpoint	Conclusions
Cox2 inhibitors^58^	Multivariate odds ratios on use or not of drug	MI and cardiac death	Rofecoxib use increases the risk of serious coronary heart disease compared with celecoxib use. Naproxen use does not protect against serious coronary heart disease
Hormone replacement therapy^59^	Logistic regression	Venous thromboembolism	Current use of hormone replacement therapy was associated with a higher risk of venous thromboembolism, although the risk seemed to be restricted to the first year of use.
Bendectin and others^60^	Pairwise comparison on mothers use of drug during pregnancy	Congenital Heart Disease	In particular, aspirin use in early pregnancy was associated with about a twofold increase in the frequency of defects in septation of the truncus arteriosus
Cox2 inhibitors^61^	Proportional hazards on use and high/low dose	MI	Rofecoxib significant effect. Aspirin reduces the effect
Appetite suppressants^62^	Pairwise comparison vs. control and frequency of event vs. drug use	Cardiac valve regurgitation	Significant effects for some of the drugs considered
Dopamine agonists^63^	Pairwise comparison of risk	Cardiac valve regurgitation	Significant effects for some drugs
Third generation oral contraceptives^64^	Pairwise comparisons	Venous thromboembolism	Risk of venous thromboembolism was slightly increased in users of third generation oral contraceptives compared with users of second generation products.
ADHD drugs in children^65^	Cox hazard ratios	Serious cardiovascular events (sudden cardiac death, acute myocardial infarction, and stroke)	No significant effect though upper CI points to doubling of events

References contained in Supplementary Material. MI, myocardial infarction.

## TRANSLATIONAL APPROACHES

**Figure 4:**
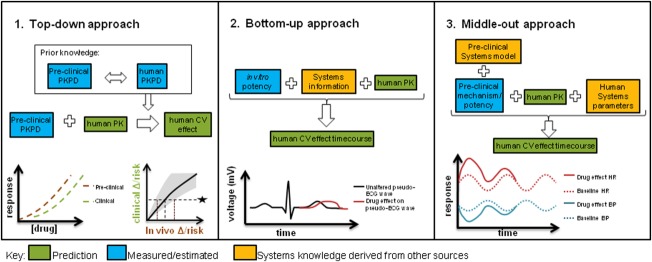
Schematic and graphical description of commonly adopted translational approaches for drug-induced CV changes and their relative use of systems information. 1. Comparative assessment between species using descriptive PK/PD modeling and simulation to identify predictive *in vitro/in vivo* assays and quantify empirical translational relationship. 2. Bottom-up systems pharmacology approach using *in vitro* potency to predict clinical effects based on mechanistic knowledge. 3. *In vivo* systems pharmacology approach to predict clinical effects based on systems knowledge and *in vivo* data.

Several types of modeling approaches have been brought to bear on cardiovascular safety, employing various levels of mechanistic insight. One of the major values of modeling for safety assessment is as translational tools to make prospective predictions about drug effects in humans. **Figure** 4 describes some of the model-based translational approaches that have been investigated to date, namely, phenomenological PK/PD methods, bottom-up systems models, and semimechanistic systems pharmacology approaches. Each of these has specific utilities and benefits in the translational context.

### Cross-species comparative assessment using descriptive PK/PD modeling and simulation, or “top-down” approaches

The traditional PK/PD framework allows quantification of the biological exposure–response relationship for better determining safe exposures. By integrating data across doses and timepoints, it can capture delayed effects, noisy data, or baseline variation over time that would be more difficult to analyze through the application of standard statistical approaches. PK/PD models have been fitted to data with multiple compounds from preclinical species (dog) and human and then the concentration–response relationship compared.^20^ They proceeded to use the relationship to predict the likely QTc change of a compound over a specific concentration range that was about to enter clinical studies; however, the concentrations required to reach 10 ms were never reached in human to fully support the prediction made. In similar cross-species comparisons^21^,^68^ the probability of reaching 10 ms was estimated for dog and human and this could also be used for prospective predictions. We noticed the reported mean slopes were ∼7–20-fold higher in human than dog, and had concerns that the dog model was less sensitive than that used in Parkinson *et al*.^20^; therefore, we adopted the Parkinson *et al*. comparative assessment as our predictive strategy for compounds showing QTc prolongation in dog studies.

By carrying out these PK/PD modeling analyses it can help to identify a pharmacokinetic driver of the safety response, for example, C_max_, AUC, or moving average of drug exposure, providing a means to potentially optimize the therapeutic index through appropriate study design.^69^ This is particularly important when effects have a slow onset (days or weeks) in comparison to daily concentration fluctuations, and the data collected over a prolonged timescale may necessitate a simplification of exposure to drive the PD model.

While a PK/PD modeling and simulation approach is valuable in making predictions about an alternative study design under similar conditions, one limitation is that they do not inherently take into account underlying physiological differences across species such as expression levels, impact of different baseline values to level of absolute change, and different turnover rates. It is likely that homeostatic mechanisms, especially for hemodynamic parameters, could obscure the underlying concentration–response relationship to different extents across species.

Incorporation of drugs with varied or even multiple mechanisms^20^ in these cross-species analyses can mitigate situations when a signal is observed preclinically and a human prediction is required, but the mechanism is unknown or not well understood.

### Bottom-up systems pharmacology model using *in vitro* potency

The bottom-up approach utilizes mechanistic knowledge of the system, allowing the input of *in vitro* data for prediction of *in vivo* effects, similar to *in vitro* to *in vivo* extrapolation (IVIVE) in PK prediction. However, purely *in vitro* systems approaches must prove their value as translational tools and part of the challenge is to understand how these scale from cellular dynamics to the whole-body system (**Figure** 2). While understanding how the single endpoint of *in vitro* inhibition compared to *in vivo* change is a standard part of the integrated preclinical risk assessment,^70^ now *in silico* predictions allow assessment of the combined effects on multiple molecular targets. This is done by combining models at a cellular level (for example, with AP models),^44^ with a multiscale approach that scales the cellular results up to the *in vivo* situation (pseudo-ECG). In the example with antipsychotics^61^ the predicted QTc effects reasonably replicated the mean observed effects. Another example predicts (through a combination of different models and techniques) the PK and PD (QTc prolongation) of domperidone (metabolized by CYP3A) and ketoconazole (CYP3A inhibitor) in a patient population.^71^ The effect of co-dosing on PK and PD was investigated, and despite having some issues predicting baseline, and some inconsistencies in the predicted and observed effects, it reproduced the study conclusions fairly well.

To date, this approach has been used to demonstrate translation largely for ion channel inhibition and QTc effects,^45^,^71^ although there is systems biology knowledge and therefore the potential for a similar approach to be applied incorporating contractility and hemodynamics. A purely *in vitro*-driven approach to predicting clinical CV changes has not yet been attempted by us but we look forward to the time when this would sit alongside the more established PK/PD approach.

### Systems pharmacology, or “middle-out” approaches

The final type of approach represents a middle-out^3^ approach that attempts to combine the best properties of the purely descriptive top-down and reductionist bottom-up approaches. For example, comprehensive *in vivo* systems pharmacology models are in existence for CV system behavior, particularly for hemodynamics,^52^,^54^,^55^ but these have so far only been implemented in a single species, making translational predictions from preclinical species to human difficult. We do not have feedback from the clinic yet to understand how successful human predictions have been and there are no reports of cross-species comparisons with these models. Alternatively, simplified model structures can be applied that capture the relevant process governing information flow without over-parameterization.^23^,^47^ Models of cardiovascular function with similar structure, but with species-specific parameters could allow for further refinement of the predictive power of these approaches. In these approaches, substituting human physiological parameters into the preclinical model is a key part of the translation. For example, when HR changes have been translated from dog to human using a PK/PD approach,^47^ the sensitivity to drug in addition to a baseline typical for human were applied. In another example where BP was translated using an indirect response model, a human baseline and rate of turnover (k_out_) for SBP and DBP was obtained from the literature and combined with a PK prediction and the drug sensitivity obtained in dog to successfully predict effects in first-time-in-human studies.^23^

In contrast to the purely phenomenological approaches, a key component of both the bottom-up and middle-out approaches is the distinction between drug-specific and physiological relevant system-specific parameters. While drug-specific pharmacodynamic parameters describe the interaction of the drug in terms of target affinity and target activation, system-specific parameters describe the processes of the biological system.^72^ Physiologically based PK models are an example that have been used in this sense and have successfully been used to make human PK predictions and estimate doses.^73^

There is great opportunity to expand on existing knowledge to bring translational understanding to the forefront of preclinical safety pharmacology assessment through a systems pharmacology approach, but there should be a balance between complexity and simplicity (what can actually be ascertained from the measurements or data available in practice). While a systems approach is preferable for translational purposes, it requires understanding or derivation of the drug effect mechanism. It must be acknowledged that when the drug effect mechanism is understood and well characterized, complex translational models can be adopted to give a robust translational prediction with certain underlying assumptions. However, when a mechanism is less understood or is a combination of effect mechanisms, it will be more challenging to make translational predictions and simpler approaches should be adopted that rely on fewer assumptions.

## DISCUSSION

With the continued pressures on increasing productivity of the R&D pipeline, M&S approaches are becoming standard practice across preclinical and clinical development, and are frequently used to analyze and translate experimental data for decision making. There exists a tremendous opportunity to leverage these approaches in the context of translational safety, particularly for cardiovascular endpoints where a deep understanding of anatomy and physiology exists across species, along with quantitative high-resolution time-course data from *in silico*, *in vitro*, preclinical, and clinical cardiovascular assays. Most of the effort in modeling drug-induced cardiovascular changes has fallen into three categories: PK/PD models of cardiovascular endpoints, systems pharmacology models such as hemodynamics, and *in vitro-*driven systems biology models. PK/PD models have allowed direct comparison across species for QTc prolongation and *in vitro-*driven systems biology models for ECG changes are coming to fruition, since the observed effects are well linked to a mechanism (ion channel inhibition) and therefore great progress has been made in this area towards reaching well-understood translation to human.

While physiologically based systems approaches for hemodynamics have been in use since the 1970s, there are very few applications of such models for understanding drug effects on the system. So far investigations of human translation have been limited, which may reflect the complexity of mathematical models required as well as the relatively recent need to reduce attrition in the clinic due to such safety issues. There are several factors that on a practical level may contribute to the limited progress in the area of hemodynamics: first, the lack of appropriate monitorable biomarkers in this complex system that potentially give rise to difficulties in parameter estimation or structural identifiability issues. Second, the investigation of effects on functional hemodynamic parameters is typically over a relatively short (24-hour) timescale, which may not allow drug-induced effects or feedback/disease processes over longer timescales to be observed or quantified in a systems model. Finally, the link to mechanism is often less clear for hemodynamic and contractility effects: the number and nature of molecular targets that could be involved is varied and therefore may be difficult to combine in an *in vitro-*driven systems model.

As mechanistic models become further advanced and gain traction in the coming years, we also expect that empirical modeling approaches will remain relevant in the safety space. This is partly because many of the known mechanisms of action for adverse events can be built into *in vitro* screens, and therefore in discovery compounds can be selected that do not interact with these targets. When effects are then observed *in vivo*, they may frequently be due to as yet unidentified mechanisms, making the use of bottom-up models more difficult.

While standard dog telemetry is the model of choice to investigate preclinical CV risk and make a quantitative translation to human, it is largely set up to investigate acute, functional, tightly PK-driven effects such as ECG effects. This type of study was originally designed for statistical analyses looking for significant differences between vehicle and compound dosed groups, and the standard designs have not changed considerably in recent years. It is typically a crossover design in which each dog receives a vehicle dose and three different doses of the drug under investigation with monitoring for up to 24 hours postdose. In order to maximize the utility of dog data there is a need for a strong, collaborative, working relationship between those conducting the study and those performing modeling on the ensuing data, to ensure that the study design is appropriate for the effect and the expected model structure if known. Further challenges to model building include incorporating the impact of feeding effects and blood sampling on the CV endpoints that can introduce unexplained or random error in a model if not appropriately accounted for.^55^ The relative sparsity of blood sampling for pharmacokinetic assessment during telemetry experiments can prove difficult when trying to build a robust pharmacokinetic model.

In addition to increased interpretability and translatability, modeling approaches can provide a strong 3R's (reduction, refinement, replacement of animal usage) benefit, as they allow for greater insight to be drawn from smaller numbers of animals and can use prediction to avoid unnecessary studies. Much discussion has taken place concerning the replacement of *in vivo* models entirely with *in silico* approaches.^74^

The most informed translation of drug-induced human changes will require knowledge of underlying physiology to be combined with systems pharmacology approaches so drug potency can be obtained from experimental data. In these scenarios, drug potency can be identified in a preclinical setting and would then be combined with human system parameters and other relevant information to make a well-validated prediction of effects in humans, and this has been shown in other areas such as myelosuppression.^75^ This approach has enormous potential to aid decision-making and risk assessment on the progression of new drugs into the clinic through their predictive capacity. Further, application of these systems models promise to increase in-depth understanding of the mechanism of drug effects when an interaction at a specific target has not yet been identified.

Going forward, it will be important that modeling with regard to CV safety focuses increased attention on CV effects such as contractility and structural damage, as efforts have been relatively concentrated on CV parameters that are readily measurable in a longitudinal manner from short-term experiments. Much of the modeling space has been dictated by the availability of data rather than the merit of CV parameters to predict long-term safety risks. The enormous interest and progress in QT has been driven by a large number of molecules entering the clinic that could be explained largely by a single mechanism. As such, the hERG channel role in QT prolongation is now much better understood and can readily be screened *in vitro*. On the other hand, cardiovascular structural effects (damage) downstream of ECG and hemodynamic changes are relatively difficult to measure and predict, tend to be chronic in nature, and arise from a greater number of potential mechanisms. These therefore represent a potentially large safety hazard. However, these effects could be modeled at least empirically through application of techniques for categorical variable modeling to preclinical cardiovascular data.

The relative rarity of cardiovascular damage in the clinic normally means that a strong safety signal will only emerge in large pivotal trials or investigations undertaken through postmarketing surveillance. At this stage this is costly to the sponsor. The challenge is therefore to quantify and predict risk of long-term CV safety issues from more frequently observed CV parameter changes.

Systems modeling may be able to help predict these chronic effects in the clinic as preclinical pathology readouts are further quantified and combined with short-term parameter changes that have successfully been modeled to date.
