# Supplementary material for: Modeling and Simulation Approaches for Cardiovascular Function and Their Role in Safety Assessment
Source: CPT Pharmacometrics Syst Pharmacol. 2015 Mar 11;4(3):e00018. doi: 10.1002/psp4.18 (PMC4394617; doi:10.1002/psp4.18)
Supplement: Supplementary file 1 — Supporting Information [file psp40004-e00018-sd1.docx]

1. Whiting B., Holford N.H., Sheiner L.B. Quantitative analysis of the disopyramide concentration-effect relationship. *Br. J. Clin. Pharmacol.* 1980, **9:** 67-75.

2. Thibonnier M., Holford N.H., Upton R.A., Blume C.D., Williams R.L. Pharmacokinetic-pharmacodynamic analysis of unbound disopyramide directly measured in serial plasma samples in man. *J. Pharmacokinet. Biopharm.* 1984, **12:** 559-573.

3. Holford N.H., Coates P.E., Guentert T.W., Riegelman S., Sheiner L.B. The effect of quinidine and its metabolites on the electrocardiogram and systolic time intervals: concentration--effect relationships. *Br. J. Clin. Pharmacol.* 1981, **11:** 187-195.

4. Karbwang J., Davis T.M., Looareesuwan S., Molunto P., Bunnag D., White N.J. A comparison of the pharmacokinetic and pharmacodynamic properties of quinine and quinidine in healthy Thai males. *Br. J. Clin. Pharmacol.* 1993, **35:** 265-271.

5. Gillis A.M., Mitchell L.B., Wyse D.G., McDonald M., Duff H.J. Quinidine pharmacodynamics in patients with arrhythmia: effects of left ventricular function. *J. Am. Coll. Cardiol.* 1995, **25:** 989-994.

6. Wang T., Bergstrand R.H., Thompson K.A., Siddoway L.A., Duff H.J., Woosley R.L.*, et al.* Concentration-dependent pharmacologic properties of sotalol. *Am. J. Cardiol.* 1986, **57:** 1160-1165.

7. Uematsu T., Kanamaru M., Nakashima M., Kanamary M. Comparative pharmacokinetic and pharmacodynamic properties of oral and intravenous (+)-sotalol in healthy volunteers. *J. Pharm. Pharmacol.* 1994, **46:** 600-605.

8. Chain A.S.Y., Krudys K.M., Danhof M., Della Pasqua O. Assessing the probability of drug-induced QTc-interval prolongation during clinical drug development. *Clin. Pharm. Ther.* 2011, **90:** 867-875.

9. Piergies A.A., Ruo T.I., Jansyn E.M., Belknap S.M., Atkinson A.J. Effect kinetics of N-acetylprocainamide-induced QT interval prolongation. *Clin. Pharm. Ther.* 1987, **42:** 107-112.

10. Shi J., Lasser T., Koziol T., Hinderling P.H. Kinetics and dynamics of sematilide. *Ther. Drug Monit.* 1995, **17:** 437-444.

11. Le Coz F., Funck-Brentano C., Morell T., Ghadanfar M.M., Jaillon P. Pharmacokinetic and pharmacodynamic modeling of the effects of oral and intravenous administrations of dofetilide on ventricular repolarization. *Clin. Pharm. Ther.* 1995, **57:** 533-542.

12. Friberg L.E., Isbister G.K., Duffull S.B. Pharmacokinetic-pharmacodynamic modelling of QT interval prolongation following citalopram overdoses. *Br. J. Clin. Pharmacol.* 2006, **61:** 177-190.

13. Dixon R., Job S., Oliver R., Tompson D., Wright J.G., Maltby K.*, et al.* Lamotrigine does not prolong QTc in a thorough QT/QTc study in healthy subjects. *Br. J. Clin. Pharmacol.* 2008, **66:** 396-404.

14. Rosignoli M.T., Di Loreto G., Dionisio P., Randomized A., Placebo D.-b., Loreto G.D. Effects of prulifloxacin on cardiac repolarization in healthy subjects: a randomized, crossover, double-blind versus placebo, moxifloxacin-controlled study. *Clin Drug Invest.* 2010, **30:** 5-14.

15. Darpo B., Bullingham R., Combs D.L., Ferber G., Hafez K. Assessment of the cardiac safety and pharmacokinetics of a short course, twice daily dose of orally-administered mifepristone in healthy male subjects. *Cardiol. J.* 2013, **20:** 152-160.

16. Maison-Blanche P., Dakhil S., Baron A., Rottey S., Millard F., Daugaard G.*, et al.* An open-label study to investigate the cardiac safety profile of cabazitaxel in patients with advanced solid tumors. *Cancer Chemother. Pharmacol.* 2014, **73:** 1241-1252.

17. Parkinson J., Visser S.a.G., Jarvis P., Pollard C., Valentin J.-P., Yates J.W.T.*, et al.* Translational pharmacokinetic-pharmacodynamic modeling of QTc effects in dog and human. *J. Pharmacol. Toxicol. Methods* 2013.

18. Dubois V., Yu H., Danhof M., Della Pasqua O., Cardiovascular Safety Project Team T.I.P.P.P. Model-based evaluation of drug-induced QT(c) prolongation for compounds in early development. *Br J Clin Pharmacol* 2014.

19. Sparve E., Quartino A.L., Luttgen M., Tunblad K., Gardlund A.T., Falting J.*, et al.* Prediction and Modeling of Effects on the QTc Interval for Clinical Safety Margin Assessment, Based on Single-Ascending-Dose Study Data with AZD3839. *J. Pharmacol. Exp. Ther.* 2014, **350**(2)**:** 469-478.

20. Lima J.J., Boudoulas H., British T., Document L., Service S., Spa B.*, et al.* Stereoselective effects of disopyramide enantiomers in humans. *J. Cardiovasc. Pharmacol.* 1987, **9:** 594-600.

21. Phillips L., Grasela T.H., Agnew J.R., Ludwig E.A., Thompson G.A. A population pharmacokinetic-pharmacodynamic analysis and model validation of azimilide. *Clin. Pharm. Ther.* 2001, **70:** 370-383.

22. Mao Z., Wheeler J.J., Townsend R., Gao Y., Kshirsagar S., Keirns J.J. Population pharmacokinetic-pharmacodynamic analysis of vernakalant hydrochloride injection (RSD1235) in atrial fibrillation or atrial flutter. *J. Pharmacokinet. Pharmacodyn.* 2011, **38:** 541-562.

23. Jonker D.M., Kenna L.a., Leishman D., Wallis R., Milligan P.a., Jonsson E.N. A pharmacokinetic-pharmacodynamic model for the quantitative prediction of dofetilide clinical QT prolongation from human ether-a-go-go-related gene current inhibition data. *Clin. Pharm. Ther.* 2005, **77:** 572-582.

24. Ohtani H., Hanada E., Yamamoto K., Sawada Y., Iga T. Pharmacokinetic-pharmacodynamic analysis of the electrocardiographic effects of terfenadine and quinidine in rats. *Biol. Pharma. Bull.* 1996, **19:** 1189-1196.

25. Ohtani H., Taninaka C., Hanada E., Kotaki H., Sato H., Sawada Y.*, et al.* Comparative pharmacodynamic analysis of Q-T interval prolongation induced by the macrolides clarithromycin, roxithromycin, and azithromycin in rats. *Antimicrob. Agents Chemother.* 2000, **44:** 2630-2637.

26. Hanada E., Ohtani H., Kotaki H., Sawada Y., Sato H., Iga T. Pharmacodynamic analysis of the electrocardiographic interaction between disopyramide and erythromycin in rats. *Journal of pharmaceutical sciences* 1999, **88**(2)**:** 234-240.

27. Ohtani H., Sato H., Iga T., Kotaki H., Sawada Y. Pharmacokinetic-pharmacodynamic analysis of the arrhythmogenic potency of a novel antiallergic agent, ebastine, in rats. *Biopharm. Drug Dispos.* 1999, **20:** 101-106.

28. Ohtani H., Odagiri Y., Sato H., Sawada Y., Iga T. A comparative pharmacodynamic study of the arrhythmogenicity of antidepressants, fluvoxamine and imipramine, in guinea pigs. *Biol. Pharma. Bull.* 2001, **24:** 550-554.

29. Minematsu T., Ohtani H., Sato H., Iga T. Pharmacokinetic/pharmacodynamic analysis of tacrolimus-induced QT prolongation in guinea pigs. *Biol. Pharma. Bull.* 1999, **22:** 1341-1346.

30. Chain A., Dubois V., Danhof M., Sturkenboom M., Della Pasqua O. Identifying the translational gap in the evaluation of drug-induced QTc-interval prolongation. *Br. J. Clin. Pharmacol.* 2013.

31. Caruso A., Frances N., Meille C., Greiter-Wilke A., Hillebrecht A., Lavé T. Translational PK/PD modeling for cardiovascular safety assessment of drug candidates: Methods and examples in drug development. *J. Pharmacol. Toxicol. Methods* 2014, **70**(1)**:** 73-85.

32. Rakhit A., Guentert T.W.W., Holford N.H.G., Verhoeven J., Riegelman S. Pharmacokinetics and pharmacodynamics of quinidine and its metabolite, quinidine-N-oxide, in beagle dogs. *Eur. J. Drug Metab. Pharmacokin.* 1983, **9:** 315-324.

33. Ollerstam A., Visser S.A.G., Persson A.H., Eklund G., Nilsson L.B., Forsberg T.*, et al.* Pharmacokinetic-pharmacodynamic modeling of drug-induced effect on the QT interval in conscious telemetered dogs. *J. Pharmacol. Toxicol. Methods* 2006, **53:** 174-183.

34. Ollerstam A., Visser S.A.G., Duker G., Forsberg T., Persson A.H., Nilsson L.B.*, et al.* Comparison of the QT interval response during sinus and paced rhythm in conscious and anesthetized beagle dogs. *J. Pharmacol. Toxicol. Methods* 2007, **56:** 131-144.

35. Nolan E.R., Feng M.R., Koup J.R., Liu J., Turluck D., Zhang Y.*, et al.* A novel predictive pharmacokinetic/pharmacodynamic model of repolarization prolongation derived from the effects of terfenadine, cisapride and E-4031 in the conscious chronic av node--ablated, His bundle-paced dog. *J. Pharmacol. Toxicol. Methods* 2006, **53:** 1-10.

36. Watson K.J., Gorczyca W.P., Umland J., Zhang Y., Chen X., Sun S.Z.*, et al.* Pharmacokinetic-pharmacodynamic modelling of the effect of Moxifloxacin on QTc prolongation in telemetered cynomolgus monkeys. *J. Pharmacol. Toxicol. Methods* 2011, **63:** 304-313.

37. Padrini R., Piovan D., Busa M., Al-Bunni M., Maiolino P., Ferrari M. Pharmacodynamic variability of flecainide assessed by QRS changes. *Clin. Pharm. Ther.* 1993, **53:** 59-64.

38. Deneer V.H.M., Lie-A-Huen L., Kingma J.H., Proost J.H., Gossen S.a., Stuurman A.*, et al.* Absorption kinetics and pharmacodynamics of two oral dosage forms of flecainide in patients with an episode of paroxysmal atrial fibrillation. *Eur. J. Clin. Pharmacol.* 2004, **60:** 693-701.

39. Fleury A., Lavé T., Jonsson F., Schmitt M., Hirkaler G., Polonchuk L.*, et al.* A pharmacokinetic–pharmacodynamic model for cardiovascular safety assessment of R1551. *J. Pharmacol. Toxicol. Methods* 2011, **63:** 123-133.

40. Sällström J., Al-Saffar A., Pehrson R. Pharmacokinetic-pharmacodynamic modeling of QRS-prolongation by flecainide: heart rate-dependent effects during sinus rhythm in conscious telemetered dogs. *J. Pharmacol. Toxicol. Methods* 2014, **69:** 24-29.

41. Jacobson I., Duker G., Florentzson M., Linhardt G., Lindhardt E., Nordkam A.K.*, et al.* Electrophysiological characterization and antiarrhythmic efficacy of the mixed potassium channel-blocking antiarrhythmic agent AZ13395438 in vitro and in vivo. *J. Cardiovasc. Pharmacol. Ther.* 2013, **18**(3)**:** 290-300.

42. Olsson R.I., Jacobson I., Bostrom J., Fex T., Bjore A., Olsson C.*, et al.* Synthesis and evaluation of diphenylphosphinic amides and diphenylphosphine oxides as inhibitors of Kv1.5. *Bioorg. Med. Chem. Lett.* 2013, **23**(3)**:** 706-710.

43. van Rijn-Bikker P.C., Ackaert O., Snelder N., van Hest R.M., Ploeger B.A., Koopmans R.P.*, et al.* Pharmacokinetic-pharmacodynamic modeling of the antihypertensive effect of eprosartan in Black and White hypertensive patients. *Clin. Pharmacokinet.* 2013, **52**(9)**:** 793-803.

44. Meredith P.A., Elliott H.L., Kelman A.W., Reid J.L. Application of Pharmacokinetic-pharmacodynamic modelling for the comparison of quinazoline alph-adrenoceptor agonists in normotensive volunteers. *J. Cardiovasc. Pharmacol.* 1985, **7:** 532-537.

45. Vincent J., Elliott H.L., Meredith P.A., Reid J.L. Doxazosin, an alpha-adrenoceptor antagonist: pharmacokinetics and concentration-effect relationships in man. *Br. J. Clin. Pharmacokin.* 1983, **15:** 719-725.

46. Keizer R.J., Gupta A., Mac Gillavry M.R., Jansen M., Wanders J., Beijnen J.H.*, et al.* A model of hypertension and proteinuria in cancer patients treated with the anti-angiogenic drug E7080. *J. Pharmacokinet. Pharmacodyn.* 2010, **37**(4)**:** 347-363.

47. Hempel G., Karlsson M.O., de Alwis D.P., Toublanc N., McNay J., Schaefer H.G. Population pharmacokinetic-pharmacodynamic modeling of moxonidine using 24-hour ambulatory blood pressure measurements. *Clin. Pharm. Ther.* 1998, **64**(6)**:** 622-635.

48. Lee J., Han S., Jeon S., Hong T., Yim D.S. Pharmacokinetic-pharmacodynamic model of fimasartan applied to predict the influence of a high fat diet on its blood pressure-lowering effect in healthy subjects. *Eur. J. Clin. Pharmacol.* 2013, **69**(1)**:** 11-20.

49. Standing J.F., Hammer G.B., Sam W.J., Drover D.R. Pharmacokinetic-pharmacodynamic modeling of the hypotensive effect of remifentanil in infants undergoing cranioplasty. *Paediatric anaesthesia* 2010, **20**(1)**:** 7-18.

50. Donnelly R., Elliott H.L., Meredith P.A., Kelman A.W., Reid J.L. Nifedipine: individual responses and concentration-effect relationships. *Hypertension* 1988, **12**(4)**:** 443-449.

51. Howgate E.M. Cross-species scaling of cardiovascular safety pharmacology using PKPD modelling and simulation. Doctor of Philosophy thesis, University of Manchester, University of Manchester, 2012.

52. Hatanaka T., Ihara K., Kodera N., Katayama K., Koizumi T. Stereoselective pharmacokinetics and pharmacodynamics of organic nitrates in rats. *J. Pharmacol. Exp. Ther.* 2001, **298**(1)**:** 346-353.

53. Sällström B., Visser S.a.G., Forsberg T., Peletier L.a., Ericson A.-C., Gabrielsson J. A pharmacodynamic turnover model capturing asymmetric circadian baselines of body temperature, heart rate and blood pressure in rats: challenges in terms of tolerance and animal-handling effects. *J. Pharmacokinet. Pharmacodyn.* 2005, **32:** 835-859.

54. Langdon G., Davis J.D., McFadyen L.M., Dewhurst M., Brunton N.S., Rawal J.K.*, et al.* Translational pharmacokinetic-pharmacodynamic modelling; application to cardiovascular safety data for PF-00821385, a novel HIV agent. *Br. J. Clin. Pharmacol.* 2010, **69**(4)**:** 336-345.

55. Diderichsen P.M., Cox E., Martin S.W., Cleton A., Ribbing J. Predicted heart rate effect of inhaled PF-00610355, a long acting beta-adrenoceptor agonist, in volunteers and patients with chronic obstructive pulmonary disease. *Br. J. Clin. Pharmacol.* 2013, **76**(5)**:** 752-762.

56. Velez de Mendizabal N., Staab A., Schafer H.G., Trommeshauser D., Doge C., Kluglich M.*, et al.* Joint population pharmacokinetic/pharmacodynamic model for the heart rate effects at rest and at the end of exercise for cilobradine. *Pharm. Res.* 2013, **30**(4)**:** 1110-1122.

57. Van der Graaf P.H., Van Schaick E.A., Mathot R.A., Ijzerman A.P., Danhof M. Mechanism-based pharmacokinetic-pharmacodynamic modeling of the effects of N6-cyclopentyladenosine analogs on heart rate in rat: estimation of in vivo operational affinity and efficacy at adenosine A1 receptors. *J. Pharmacol. Exp. Ther.* 1997, **283**(2)**:** 809-816.

58. Graham D.J., Campen D., Hui R., Spence M., Cheetham C., Levy G.*, et al.* Risk of acute myocardial infarction and sudden cardiac death in patients treated with cyclo-oxygenase 2 selective and non-selective non-steroidal anti-inflammatory drugs: nested case-control study. *Lancet* 2005, **365**(9458)**:** 475-481.

59. Gutthann S., Rodriguez L., Castellsague J., LOliart A. Hormone replacement therapy and risk of venous thromboembolism:population based case-control study. *Br. Med. J.* 1997, **314:** 796-800.

60. Zierler S., Rothman K. Congenital heart disease in relation to maternal use of bendectin and other drugs during pregnancy. *New Eng. J. Med.* 1985, **313:** 347-352.

61. Levesque L., Brophy J., Zhang B. The risk for myocardial infarction with cyclooxygenase-2 inhibitors: a population study of elderly adults. *Ann. Int. Med.* 2005, **142:** 481-489.

62. Jick H., Vasilakis C., Weinrauch L., Meier C., Jick S., Derby L. A population-based study of appetite-suppressant drugs and the risk of cardiovascular regurgitation. *New Eng. J. Med.* 1998, **339:** 719-724.

63. Schade R., Andersohn F., Suissa S., Haverkamp W., Garbe E. Dopamine agonists and the risk of cardiac-valve regurgitation. *New Eng. J. Med.* 2002, **2007**(356)**:** 29-38.

64. Spitzer W., Lewis M., Heinemann L., Thorogood M., MacRae K. Third generation oral contraceptives and risk of venous thromboembolic disorders: an interational case-control study. *Br. Med. J.* 1996, **312**(83-88).

65. Cooper W., Habel L., Sox C., Chan K., Arbogast P., Cheetham T.*, et al.* ADHD Drugs and serious cardiovascular events in children and young adults. *New Eng. J. Med.* 2011, **365:** 1896-1904.

66. Francheteau P., Steimer J.L., Merdjan H., Guerret M., Dubray C. A mathematical model for dynamics of cardiovascular drug action: application to intravenous dihydropyridines in healthy volunteers. *J. Pharmacokinet. Biopharm.* 1993, **21:** 489-514.

67. Snelder N., Ploeger B.a., Luttringer O., Rigel D.F., Webb R.L., Feldman D.*, et al.* PKPD modeling of the interrelationship between mean arterial blood pressure, cardiac output and total peripheral resistance in conscious rats. *Br. J. Pharmacol.* 2013, **169:** 1510-1524.

68. Snelder N., Ploeger B.A., Luttringer O., Rigel D.F., Fu F., Beil M.*, et al.* Drug effects on the cardiovascular system in conscious rats - separating cardiac output into heart rate and stroke volume using PKPD modeling. *Br. J. Pharmacol.* 2014, **In press**.

69. Upton R.N., Ludbrook G.L. Pharmacokinetic-pharmacodynamic modelling of the cardiovascular effects of drugs - method development and application to magnesium in sheep. *BMC Pharmacol.* 2005, **5:** 5.

70. Ottesen J.T., Novak V., Olufsen M.S. Development of patient specific cardiovascular models predicting dynamics in response to orthostatic stress challenges. 2009**:** 1-45.
